# Do preoperative depressive symptoms predict quality of life after laparoscopic cholecystectomy: A longitudinal prospective study

**DOI:** 10.1371/journal.pone.0202266

**Published:** 2018-08-30

**Authors:** Hao-Hsien Lee, Chong-Chi Chiu, King-Teh Lee, Jhi-Joung Wang, Jin-Jia Lin, Chien-Ming Chao, Hon-Yi Shi

**Affiliations:** 1 Department of General Surgery, Chi Mei Medical Center, Liouying, Taiwan; 2 Department of General Surgery, Chi Mei Medical Center, Yongkang, Taiwan; 3 Department of Electrical Engineering, Southern Taiwan University of Science and Technology, Tainan, Taiwan; 4 Division of Hepatobiliary Surgery, Department of Surgery, Kaohsiung Medical University Hospital, Kaohsiung, Taiwan; 5 Department of Healthcare Administration and Medical Informatics, Kaohsiung Medical University, Kaohsiung, Taiwan; 6 Department of Medical Research, Chi Mei Medical Center, Tainan, Taiwan; 7 Department of Psychiatry, Chi-Mei Medical Center, Yongkang, Tainan, Taiwan; 8 Department of Psychiatry, Chi-Mei Hospital, Liouying, Tainan, Taiwan; 9 Department of Psychiatry, School of Medicine, College of Medicine, Taipei Medical University, Taipei, Taiwan; 10 Department of Intensive Care Medicine, Chi Mei Medical Center, Liouying, Taiwan; 11 Department of Business Management, National Sun Yat-sen University, Kaohsiung, Taiwan; 12 Department of Medical Research, Kaohsiung Medical University Hospital, Kaohsiung, Taiwan; University Hospital Tübingen, GERMANY

## Abstract

**Objective:**

The impact of preoperative depressive symptoms on quality of life (QOL) after laparoscopic cholecystectomy (LC) remains unclear. The purpose of this study was to develop a benchmark for capturing the burden of depressive symptoms on QOL after LC and for supporting evidence-based clinical interventions for remediating these effects.

**Methods:**

Patients diagnosed with depressive symptoms (Beck Depression Inventory score > 13) after LC (n = 336) were classified into a depressive symptoms group. Propensity score matching was then used to match them with 336 patients in a non-depressive symptoms group for all potential confounding factors. All patients completed the 36-item Short Form Health Survey (SF-36) and the Gastrointestinal Quality of Life Index (GIQLI) at baseline and at 2 years postoperatively. The 95% confidence intervals (CIs) for differences in responsiveness estimates were derived by bootstrap estimation.

**Results:**

The GIQLI results revealed that the non-depressive symptoms group had relatively stronger responses for emotional impairment (4.10, 95% CI 2.81 to 5.39) and social impairment (4.06, 95% CI 2.65 to 5.46) in comparison with the depressive symptoms group. In the SF-36, the non-depressive symptoms group also had stronger responses for role emotional (12.63, 95% CI 10.73 to 14.54), social functioning (11.25, 95% CI 9.85 to 12.65), vitality (3.81, 95% CI 2.82 to 4.81), mental health (11.97, 95% CI 10.36 to 13.56) and general health (3.84, 95% CI 2.95 to 4.75).

**Conclusions:**

Depressive symptoms complicate the management of LC patients and are associated with poorer outcomes. Because depressive symptoms are very common, further studies are needed to evaluate integrated and comprehensive approaches for assessing and treating these symptoms.

## Introduction

Laparoscopic cholecystectomy (LC) is of particular interest as it is a common surgical procedure in which the propensity for full recovery shows wide individual variation, even in the absence of complications. For example, a single-blind and randomized controlled trial cohort study reported that 0.9% of LC patients have severe anxiety or depression within 1 year after surgery whereas up to 15% of LC patients have mild depression within 1 year after surgery [1].

Depressive symptoms are the main contributors to the global burden of neuropsychiatric disease and are associated with increased suicide risk, increased health-care costs, and decreased economic productivity [2–4]. The impact of depressive symptoms is often estimated in terms of clinical endpoints such as risk of complications, probability of readmission, and probability of survival. These measures are essential but do not fully capture the impact of depressive symptoms in terms of patient-reported outcomes such as quality of life (QOL) [5–7]. Therefore, researchers have attempted to use QOL assessments to improve understanding of the burden of depressive symptoms and to evaluate the effects of medical treatments on depression.

In recent years, researchers have begun using QOL as a primary outcome measure because it provides a complex multi-dimensional assessment of physical, psychological and social well-being. For example, symptoms of various diseases and adverse effects of their treatments are associated with reductions in QOL [8, 9]. Postoperative mental health is also highly relevant to surgical outcomes. Accurately identifying and monitoring patients at risk of developing mental health issues is also expected to have positive effects on outcomes of targeted surgical interventions [10, 11]. Nevertheless, no studies have specifically studied how depression affects QOL after LC.

Therefore, this longitudinal prospective study analyzed QOL changes in patients diagnosed with depressive symptoms within 2 years after undergoing LC. Over a similar time frame, QOL changes were then compared between the enrolled subjects and a propensity score-matched control group drawn from the same population base but without depressive symptoms. The objectives of this study were to provide a benchmark for capturing the effects of preoperative depressive symptoms on QOL after LC, to improve understanding of these effects, and to develop evidence-based clinical interventions for remediating these effects.

## Materials and methods

### Study design and population

**Fig 1:**
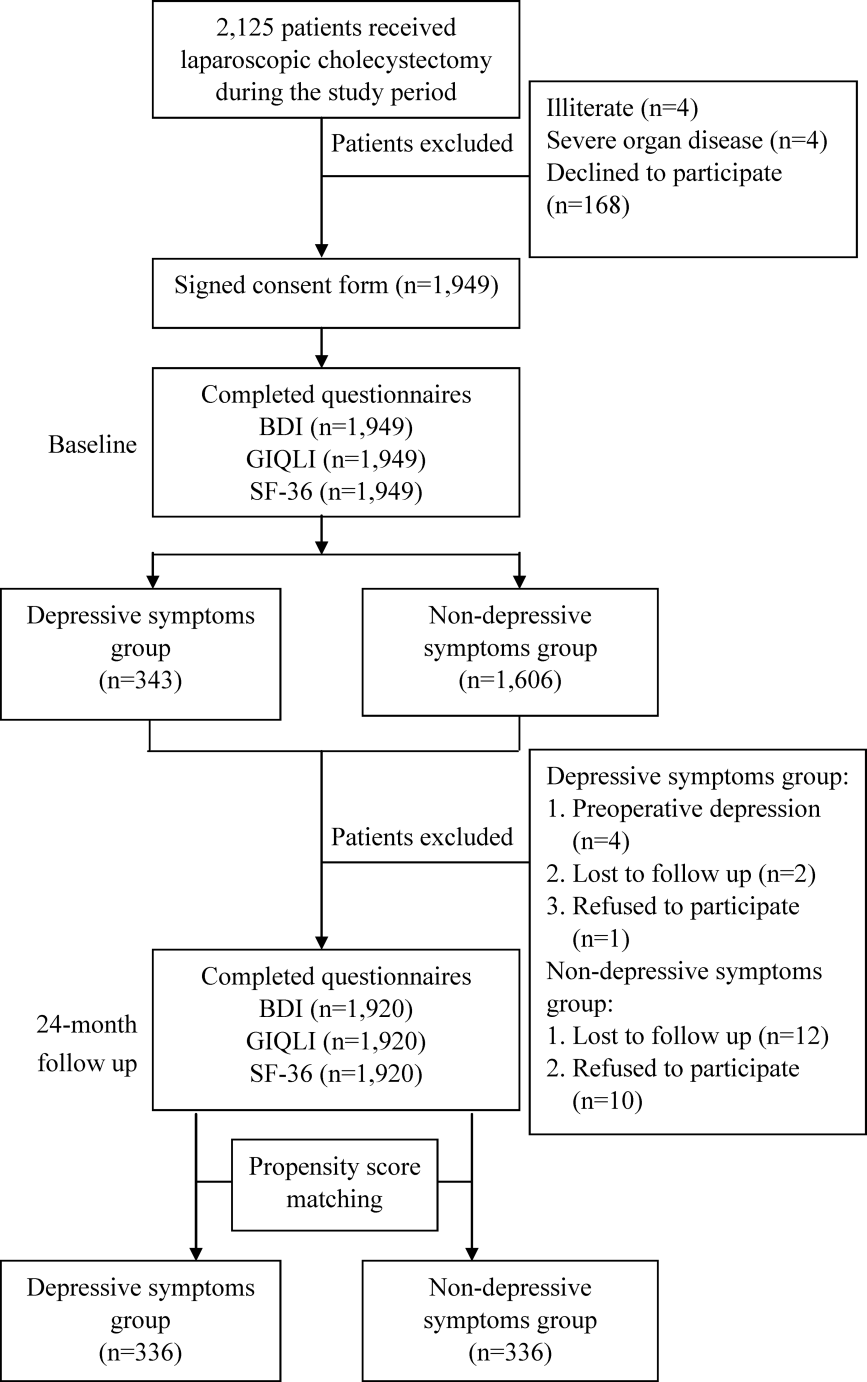
Flow chart of recruitment and study procedure. GIQLI: Gastrointestinal Quality of Life Index, SF-36: 36-item Short-Form Health Survey.

All patients who had undergone LC performed between March, 2010 and October, 2014 by any one of three senior surgeons (KT, HH, CC) practicing at two tertiary academic hospitals in southern Taiwan were surveyed with three instruments: the Chinese version of the Beck Depression Inventory (BDI) [12], the 36-item Short Form Health Survey (SF-36) [13] and the Gastrointestinal Quality of Life Index (GIQLI) [14]. The institutional review board of Kaohsiung Medical University Hospital approved this prospective study. The diagnostic codes included in the International Classification of Diseases, 9th Revision, Clinical Modification were used to screen data contained in the medical records of the participants. The enrollment criteria in this study were records of codes for primary or secondary diagnosis of gallbladder stones, gallbladder polyps, or acute cholecystitis (codes 574.00 to 576.99) and a code for LC performed as a primary or secondary procedure (code 51.23). Fig 1 shows that 2,125 LC patients were initially recruited for the study. Exclusion criteria were illiteracy (n = 4) and severe organ disease (n = 4). At baseline, 1,949 eligible subjects gave written informed consent to participate. Of these, 29 were excluded: four who had preoperative depression, 14 who were lost to follow up, and 11 who declined to participate. All 1,920 of the remaining LC subjects were enrolled in the study and completed the preoperative and 2-year postoperative assessments. All LC patient characteristics were matched between individuals with and without depressive symptoms. The caliper matching method was used for one-to-one matching between the depressive symptoms group and the non-depressive symptoms group based on propensity scores.

### Instruments and measurements

Depressive symptoms were measured with version II of the BDI. Total scores ranged from 0 to 63 points, where high scores indicate severe depressive symptoms. The BDI score was dichotomized at the clinical reference threshold for mild depression (> 13). The Chinese version of the measure has shown good reliability and validity [15].

The score for the SF-36 used to measure QOL outcomes was used as a dependent variable. This widely-used measure of generic QOL includes 36 items for evaluating physical functioning, role-physical, role-emotional, social functioning, bodily pain, vitality, mental health, and general health. Each SF-36 subscale score was converted to a score from 0 to 100 representing low to high QOL, respectively. A translated version of the SF-36 has been validated in Chinese populations [13].

The GIQLI is recognized as a valid and reliable instrument for measuring QOL, especially in patients undergoing cholecystectomy [14, 16]. Its 36 items include symptomatology (19 items), emotional impairment (5 items), physical impairment (7 items), social impairment (4 items) and medical treatment effects (1 item). Each item is scored from 0 to 4 with a higher score indicating a better QOL. The total GIQLI score ranges from 0 to 144. A Chinese version of the GIQLI has demonstrated acceptable validity [16].

The following patient data obtained by records reviews and questionnaire surveys were tested as independent variables; age, gender, body mass index (BMI), education, Charlson co-morbidity index (CCI) score, marital status, previous abdominal surgery, surgical factors, patient referral source, current alcohol or tobacco use, preoperative functional status, operating time, American Society of Anesthesiologist (ASA) score, current complications, operation time, length of stay (LOS) and re-hospitalization within 30 days.

### Statistical analysis

The unit of analysis in this study was the individual LC patient. To minimize the potential effects of confounding factors when comparing QOL between the case patients with depressive symptoms and the control subjects without these symptoms, control subjects were matched to case patients by performing propensity score matching (PSM) procedures as described in the literature [17]. Specifically, the PSM method was used to perform one-to-one matching between the case patients and control subjects based on age, gender, BMI, education, CCI score, marital status, previous abdominal surgery, surgical factors, patient referral source, current alcohol or tobacco use, preoperative functional status, operating time, ASA score, current complications, operation time, LOS, and re-hospitalization within 30 days. Of the 1,920 patients analyzed, 336 patients who had depressive symptoms immediately before LC were classified into the depressive symptoms group. Another 336 patients matched by PSM were classified into the non-depressive symptoms group.

The relative magnitude of change between two time intervals was assessed by calculating effect size (ES), which is the difference between the mean scores for two time intervals divided by the standard deviation in the previous (or formal) time-interval score [18]. Effect sizes of 0.2, 0.5 and 0.8 are typically interpreted as small, medium and large changes, respectively [18]. The bootstrap method was used to calculate the 95% confidence intervals (CIs) for ES estimates of each subscale of the SF-36 and the GIQLI and to test for significant differences in ES estimates between the two groups [19]. One thousand bootstrap samples were used to calculate group differences in ES estimates for each subscale of the SF-36 and the GIQLI. The 95% CIs for the differences in ES estimates between these two groups were also calculated.

Additionally, a sensitivity analysis of 2-year postoperative QOL in LC patients with depressive symptoms (n = 336) and whole control subjects without depressive symptoms (n = 1,584) were conducted to evaluate whether the result of the propensity matching approach is the same as the result of the whole sample approach. Statistical analyses were performed using STATISTICA 10.0 (StatSoft, Tulsa, OK). All tests were two-sided, and p values less than 0.05 were considered statistically significant.

## Results

Table 1 compares patient characteristics between the depressive symptoms group and the matched non-depressive symptoms group. After propensity score matching, all the study covariates were similar at baseline between the two groups.

**Table 1:** Laparoscopic cholecystectomy patients with depressive symptoms in comparison with matched control subjects without depressive symptoms.

Characteristics		Before PSM			After PSM	
	Depressive symptoms (n = 336)		Non-depressive symptoms (n = 1,584)	P value	Non-depressive symptoms (n = 336)	P value
Mean age (SD), years	55.3 (14.8)		54.8 (14.9)	<0.001	55.7 (14.3)	0.645
Body mass index (SD), kg/m^2^	24.7 (3.6)		23.7 (4.0)	<0.001	24.9 (3.8)	0.927
Duration of symptoms (SD), months	11.7 (23.7)		12.1 (24.6)	<0.001	11.9 (24.3)	0.633
CCI (SD), score	1.0 (1.0)		1.0 (1.2)	0.666	1.0 (1.0)	0.903
Gender, %						
Male	57.3		55.9	0.546	57.4	0.976
Female	42.7		44.1		42.6	
Education, %						
No formal education/primary school	34.9		31.8	0.961	34.5	0.993
Junior high school	14.3		13.8		14.3	
Senior high school	30.0		31.4		30.2	
College	20.8		23.0		21.0	
Marital status, %						
Single	9.3		10.9	0.904	9.3	1.000
Married	90.7		89.1		90.7	
Previous abdominal surgery, %						
Yes	32.5		33.4	0.892	32.3	0.976
No	67.5		66.6		67.7	
Surgical factors, %						
Symptomatic gallstones	62.9		61.6	0.951	63.0	0.988
Acute cholecystitis with gallstones	37.1		38.4		37.0	
Patient referral source, %						
Outpatient department	75.8		77.0	0.842	75.8	1.000
Emergency department	24.2		23.0		24.2	
Current drinker, %						
Yes	12.3		14.0	0.722	12.3	1.000
No	87.7		86.0		87.7	
Current smoker, %						
Yes	17.0		15.3	0.744	17.0	1.000
No	83.0		84.7		83.0	
Length of stay (SD), days	2.8 (2.6)		2.5 (2.4)	<0.001	2.7 (2.6)	0.440
Re-hospitalization within 30 days, %						
Yes	3.0		4.1	0.968	3.0	1.000
No	97.0		95.8		97.0	
Current complications, %						
No	93.4		94.6	0.721	93.4	1.000
Yes	6.6		5.4		6.6	
Operation time (SD), minutes	85.8 (45.5)		82.3 (43.0)	<0.001	85.5 (45.2)	0.425
ASA (SD), score	2.2 (0.5)		2.7 (0.6)	<0.001	2.2 (0.5)	1.000

PSM, propensity score matching; SD, standard deviation; CCI, Charlson co-morbidity index; ASA, American society of anesthesiologists

Table 2 compares the ES values for the two groups from baseline to 2 years after diagnosis of depressive symptoms. For each GIQLI subscale, the ES values were larger in the non-depressive symptoms group than in the depressive symptoms group. However, ES values for GIQLI emotional impairment and social impairment for were much larger in the non-depressive symptoms group than in the depressive symptoms group. Differences were considered statistically significant at the 0.05 level for CIs other than zero. During the same period, responses for both GIQLI emotional impairment (4.10, 95% CI 2.81 to 5.39) and social impairment (4.06, 95% CI 2.65 to 5.46) were stronger in the non-depressive symptoms group compared to the depressive symptoms group.

**Table 2:** Adjusted changes and effect size in subscales of the gastrointestinal quality of life index (GIQLI) for laparoscopic cholecystectomy (LC) patients with depressive symptoms and matched control subjects without depressive symptoms: comparisons at baseline (time 1) and at 2 years after depressive symptoms diagnosis (time 2)^a^.

	Depressive symptoms group (n = 336)			Matched non-depressive symptoms group (n = 336)			Mean difference^b^ (estimate [95% CI])
Subscales	Mean score (SD) at baseline	Mean score (SD) at 2 years after depressive symptoms diagnosis	Effect size (time 2 vs. time 1)	Mean score (SD) at baseline	Mean score (SD) at 2 years after depressive symptoms diagnosis	Effect size (time 2 vs. time 1)	
Symptomatology	61.18 (3.24)	70.05 (4.81)	2.73	62.63 (3.35)	72.47 (3.69)	2.94	0.11 (-0.20, 0.43)
Emotional impairment	14.63 (2.49)	10.34 (2.11)	-1.72	14.28 (1.95)	18.92 (2.32)	2.38	4.10 (2.81, 5.39)
Physical impairment	17.16 (1.37)	20.69 (2.71)	2.58	18.47 (1.89)	23.53 (2.84)	2.68	0.09 (-0.03, 0.21)
Social impairment	10.81 (1.31)	8.42 (1.60)	-1.82	10.79 (1.82)	14.86 (1.27)	2.24	4.06 (2.65, 5.46)

^a^Change scores were adjusted for characteristics of LC patients. To derive 95% CIs, standard errors were adjusted for clustering effects due to health plan and matched design for the different time periods of the study. A high score indicates a high quality of life. The mean change in each GIQLI subscale score exceeded the minimal clinically important difference of one-half of a standard deviation.

^b^Mean difference (matched non-depressive symptoms group–depressive symptoms group) is presented as effect size (95% CI) obtained by bootstrapping.

SD, standard deviation; CI, confidence interval.

Table 3 further shows that the ES of each SF-36 subscale score from baseline to 2 years after diagnosis was larger in the non-depressive symptoms group than in the depressive symptoms group. However, the ES of scores for the SF-36 role emotional, social functioning, vitality, mental health and general health for the same period were much larger in the non-depressive symptoms group than in the depressive symptoms group. During the same period, the non-depressive symptoms patients revealed relatively stronger responses for SF-36 role emotional (12.63, 95% CI 10.73 to 14.54), social functioning (11.25, 95% CI 9.85 to 12.65), vitality (3.81, 95% CI 2.82 to 4.81), mental health (11.97, 95% CI 10.36 to 13.56) and general health (3.84, 95% CI 2.95 to 4.75). Additionally, it shows the result of the propensity matching approach is the same as the result of the whole sample approach (Table 4).

**Table 3:** Adjusted changes and effect size in the 36-item short-form health survey (SF-36) subscales for laparoscopic cholecystectomy (LC) patients with depressive symptoms and matched control subjects without depressive symptoms: comparisons at baseline (time 1) and at 2 years after depressive symptoms diagnosis (time 2)^a^.

	Depressive symptoms group (n = 336)			Matched non-depressive symptoms group (n = 336)			Mean difference^b^ (estimate [95% CI])
Subscales	Mean score (SD) at baseline	Mean score (SD) at 2 years after depressive symptoms diagnosis	Effect size (time 2 vs. time 1)	Mean score (SD) at baseline	Mean score (SD) at 2 years after depressive symptoms diagnosis	Effect size (time 2 vs. time 1)	
Physical functioning	78.65 (1.32)	90.81 (2.64)	9.21	78.48 (1.37)	92.16 (1.88)	9.98	0.77 (-0.24, 1.77)
Role physical	56.73 (2.45)	88.26 (6.75)	12.87	57.16 (2.56)	92.22 (2.06)	13.06	0.19 (-1.11, 1.49)
Role emotional	69.34 (4.62)	52.28 (6.68)	-3.69	66.09 (2.77)	91.46 (2.42)	8.93	12.63 (10.73, 14.54)
Social functioning	81.85 (3.07)	60.16 (2.11)	-7.07	82.41 (2.62)	91.85 (2.31)	4.18	11.25 (9.85, 12.65)
Bodily pain	52.30 (3.32)	90.36 (1.89)	11.46	57.67 (2.78)	92.36 (2.20)	12.15	0.69 (-0.41, 1.79)
Vitality	58.74 (2.68)	78.16 (3.75)	7.25	59.13 (2.16)	88.81 (2.41)	11.06	3.81 (2.82, 4.81)
Mental health	70.37 (2.45)	60.91 (1.71)	-3.86	71.48 (1.19)	87.33 (2.94)	8.11	11.97 (10.36, 13.56)
General health	59.06 (2.50)	68.31 (3.32)	3.70	57.54 (2.31)	73.25 (1.91)	7.54	3.84 (2.95, 4.75)

^a^Change scores were adjusted for characteristics of LC patients. To derive the 95% CIs, standard errors were adjusted for clustering effects due to health plan and matched design for the different time periods of the study. A high score indicates a high QOL. The mean change in each SF-36 subscale exceeded the minimal clinically important difference of one-half of a standard deviation.

^b^Mean difference (matched non-depressive symptoms group–depressive symptoms group) is presented as effect size (95% CI) obtained by bootstrapping).

SD, standard deviation; CI, confidence interval.

**Table 4:** The coefficients (standard deviation) of multiple linear regression analyses of 2-year postoperative quality of life in laparoscopic cholecystectomy patients with depressive symptoms (n = 336) and whole control subjects without depressive symptoms (n = 1,584).

Variable	GIQLI				SF-36							
	SYM	EMO	PHY	SOC	PF	RP	RE	SF	BP	VT	MH	GH
Without depressive symptoms vs. depressive symptoms (reference)	1.84 (4.09)	2.85*** (0.53)	1.25 (1.13)	1.65*** (0.46)	2.89 (2.47)	3.07 (4.03)	3.24*** (0.31)	3.01*** (0.29)	1.01 (3.65)	1.36** (0.41)	2.99*** (0.36)	1.57** (0.42)

GIQLI, gastrointestinal quality of life index; SYM, Symptomatology; EMO, emotional impairment; PHY, physical impairment; SOC, social impairment; PF, physical functioning; RP, role-physical; RE, role-emotional; SF, social functioning; BP, bodily pain; VT, vitality; MH, mental health; GH, general health

*P<0.05

**P<0.01

***P<0.001

## Discussion

This longitudinal long-term population-based study examined the impact of depressive symptoms on QOL after LC. Notably, the two groups had similar scores on all QOL subscales before the time at which depressive symptoms had been diagnosed in the depressive symptoms group. After diagnosis of depressive symptoms, the depressive symptoms group showed a substantial decrement in QOL relative to the non-depressive symptoms group. To our knowledge, this study is the first longitudinal study to measure long-term changes in the QOL of LC patients before and after a diagnosis of depressive symptoms in comparison with a propensity-score matched control group of LC patients without depressive symptoms. Thus, the findings of this study improve understanding of the impact of depressive symptoms and treatment on the physical, mental, and social well-being of LC patients and their functional status.

Baker et al used a battery of instruments for psychological assessment of 51 patients with documented gastroesophageal reflux disease (GERD) and in 43 age-matched controls [20]. They suggested that, although most GERD patients were psychologically similar to the controls, the GERD patients were more likely to include a subset of patients with psychological distress (including depression, somatization, anxiety, and abnormally high intensity of distress symptoms, n = 15). Kamolz et al evaluated the outcomes of laparoscopic antireflux surgery in patients with known anxiety symptoms [21]. They observed that some patients showed minimal relief of depressive symptoms even when the surgery improved their QOL and corrected their acid reflux. In Biertho et al, patients treated with laparoscopic Nissen fundoplication for GERD (n = 17) were compared to a control group of patients treated with elective LC for biliary colic (n = 10) [22]. Psychological assessments were performed before surgery and at 3 and 6 months after surgery. Although psychological assessment results did not significantly differ between the GERD group and the control group, a subset of patients with ongoing GERD symptoms had significantly higher somatization levels compared to the rest of the GERD group. These results suggest that psychological factors have a role in various gastrointestinal illnesses, but their impacts on surgical outcomes such as patient-reported QOL after LC need further study. In the LC patients in the present study, depressive symptoms had long-term impacts on QOL whether they occurred simultaneously or independently.

In the current study, 343 (17.60%) of the 1,949 patients had a principal diagnosis of depressive symptoms immediately before LC, and the prevalence of depressive symptoms after LC (24%) was lower than that reported in previous studies [22, 23]. Outpatient LC has proven safe and effective even for older and high-risk patients undergoing elective operations. However, the most common problems in ambulatory surgical patients are postoperative pain, nausea and vomiting. Depression is reportedly lower in patients who undergo LC followed by an overnight hospital stay compared to those who undergo LC followed by a same-day discharge, probably due to concerns about complications and pain occurring at home without immediate access to medical care [24]. The LOS for LC is longer in Taiwan populations than in western populations, possibly due to different treatment protocols or due to cultural differences resulting in different treatment decisions. The relatively shorter LOS observed in patients who undergo LC in the United States may explain their higher prevalence rate of depressive symptoms after LC.

The findings of the study show the importance of assessing the psychological well-being of the LC patient population. Patients in Taiwan may tend to avoid openly expressing their emotions because the local culture emphasizes tolerance and harmonious interpersonal relationships [24]. Therefore, including depressive symptoms in assessments of LC outcomes is vital. To enhance QOL after LC, patients who exhibit depressive symptoms should be promptly referred to a clinical psychiatrist or psychologist for further treatment.

This study has several important advantages over earlier prospective studies of QOL change, which typically capture baseline status before initiation of therapy but after diagnosis of depressive symptoms [25–27]. Since the impact of a depression symptom diagnosis on the health and well-being of a patient can contaminate a baseline assessment, baseline QOL should ideally be measured before the diagnosis [25–27]. This study provides valuable information not only for researchers who are interested in future descriptive and mechanistic studies, but also for clinicians providing care for LC patients. The empirical data obtained in this study indicate the potential negative impact of these symptoms on QOL in all LC patients and validate what many clinicians intuitively know about these symptoms. However, they advance our knowledge by quantifying these effects and providing an evidence base for future research and clinical interventions aimed at understanding and remediating depressive symptoms.

This study showed that depressive symptoms have significant associations with emotional impairment and social impairment in the GIQLI and with role emotional, social functioning, vitality, mental health and general health in the SF-36. These findings are consistent with Mystakidou et al. [28], who reported that emotional function is significantly associated with depression, and with Shim et al. [29], who showed that depression was the most important psychological factor in the QOL of breast cancer patients in Germany, Japan, and South Korea. Interestingly, the cross-cultural comparison in Shim et al. further showed that, in German and Japanese patients, QOL was substantially decreased by depression; in South Korean patients, however, QOL was not decreased by depression or other stress factors such as anxiety and post-traumatic stress symptoms [29].

The current study collected data for LC surgery patients who had been under the supervision of one of three surgeons in two different medical centers. Each surgeon had performed the highest volume of LC surgery procedures in his respective hospital during the previous two years. This sample selection procedure ensured that patient outcome data would not be affected by surgeons with limited experience. By focusing the analysis on procedures performed by these four surgeons, the results of this study are more representative of all LC patients compared to one analyzing those performed by a single surgeon. Several important findings of this study improve understanding of the burden of depressive symptoms among LC patients, which is particularly important given that depressive symptoms are the most common mental health problems in surgical patients and that these symptoms are often unrecognized and/or untreated.

A major limitation of this study is the reliance on self-reported BDI for assessment of depressive symptoms. Although all assessment tools were evidence-based reported symptom levels are uncertain, and some clinician-rated scales may yield different conclusions [30]. Further, the history of a specific depression disorder in some LC patients may have been associated with unique BDI trajectories. To address these issues, future studies should perform validated structured clinical interviews specifically for assessing depression disorders. Future research should also consider pre-existing depression disorders in evaluations of the impact of depressive symptoms on QOL.

## Conclusions

Since patients who undergo routine LC rarely reveal depressive symptoms unless questioned, most studies of LC have focused on technical aspects of the procedure. Therefore, this study examined the psychological status of LC patients during routine follow-up examinations and evaluated their need for professional psychiatric or psychological treatment. Inclusion of psychological status in routine follow up may reduce the incidence of depressive symptoms in LC patients and may improve their QOL and coping abilities. The above factors should be considered when planning rehabilitation and social re-adaptation interventions. Because depressive symptoms further complicate the management of LC patients and are associated with poorer outcomes, further studies are needed to test integrated and comprehensive approaches for assessing and treating these symptoms.

## Supporting information

S1 Table Parts of data sets used for evaluating the impact of preoperative depressive symptoms on the gastrointestinal quality of life index subscales scores after laparoscopic cholecystectomy (LC). (DOC)

S2 Table Parts of data sets used for evaluating the impact of preoperative depressive symptoms on the SF-36 subscales scores after laparoscopic cholecystectomy. (DOC)
