# Supplementary material for: Do preoperative depressive symptoms predict quality of life after laparoscopic cholecystectomy: A longitudinal prospective study
Source: PLoS One. 2018 Aug 30;13(8):e0202266. doi: 10.1371/journal.pone.0202266 (PMC6116980; doi:10.1371/journal.pone.0202266)
Supplement: S1 Table — (DOC) [file pone.0202266.s001.doc]

**Appendix S1**

Parts of data sets used for evaluating the impact of preoperative depressive symptoms on the gastrointestinal quality of life index subscales scores after laparoscopic cholecystectomy (LC).

| ID | Symptomatology_  baseline | Emotional impairment_  baseline | Physical impairment_  baseline | Social  Impairment_  baseline | Symptomatology_  2^nd^ year | Emotional impairment_  2^nd^ year | Physical impairment_  2^nd^ year | Social  Impairment_  2^nd^ year | Depressive  symptoms^*^ |
| --- | --- | --- | --- | --- | --- | --- | --- | --- | --- |
| 1 | 52 | 9 | 14 | 8 | 66 | 16 | 22 | 11 | 0 |
| 2 | 51 | 10 | 15 | 8 | 67 | 17 | 23 | 12 | 0 |
| 3 | 62 | 13 | 16 | 9 | 72 | 18 | 23 | 13 | 0 |
| 4 | 67 | 10 | 17 | 9 | 74 | 19 | 28 | 16 | 0 |
| 5 | 66 | 9 | 18 | 10 | 72 | 19 | 25 | 15 | 0 |
| 6 | 65 | 14 | 21 | 10 | 76 | 20 | 20 | 16 | 0 |
| 7 | 63 | 16 | 21 | 11 | 76 | 20 | 28 | 16 | 0 |
| 8 | 50 | 8 | 11 | 6 | 58 | 9 | 15 | 6 | 1 |
| 9 | 54 | 7 | 12 | 7 | 62 | 11 | 16 | 6 | 1 |
| 10 | 58 | 10 | 14 | 8 | 68 | 13 | 17 | 7 | 1 |
| 11 | 63 | 11 | 15 | 9 | 68 | 8 | 20 | 8 | 1 |
| 12 | 62 | 12 | 18 | 10 | 73 | 13 | 22 | 8 | 1 |
| 13 | 64 | 13 | 19 | 10 | 76 | 12 | 23 | 6 | 1 |
| 14 | 65 | 14 | 20 | 12 | 76 | 12 | 20 | 8 | 1 |

^*^0=non-depressive symptoms group; 1=depressive symptoms group
