# Supplementary material for: Do preoperative depressive symptoms predict quality of life after laparoscopic cholecystectomy: A longitudinal prospective study
Source: PLoS One. 2018 Aug 30;13(8):e0202266. doi: 10.1371/journal.pone.0202266 (PMC6116980; doi:10.1371/journal.pone.0202266)
Supplement: S2 Table — (DOC) [file pone.0202266.s002.doc]

Appendix S2

Parts of data sets used for evaluating the impact of preoperative depressive symptoms on the SF-36 subscales scores after laparoscopic cholecystectomy.

| ID | PF_  baseline | RP_  baseline | RE_  baseline | SF_  baseline | BP_  baseline | VT_  baseline | MH_  baseline | GH_  baseline | PF_  2^nd^ year | RP_  2^nd^ year | RE_  2^nd^ year | SF_  2^nd^ year | BP_  2^nd^ year | VT_  2^nd^ year | MH_  2^nd^ year | GH_  2^nd^ year | Depressive  symptoms^*^ |
| --- | --- | --- | --- | --- | --- | --- | --- | --- | --- | --- | --- | --- | --- | --- | --- | --- | --- |
| 1 | 45 | 25 | 33.3 | 37.5 | 51 | 45 | 52 | 25 | 85 | 83 | 85 | 87.5 | 84 | 70 | 84 | 70 | 0 |
| 2 | 50 | 50 | 33.3 | 50 | 51 | 45 | 64 | 62 | 85 | 83 | 98 | 94.7 | 84 | 70 | 84 | 67 | 0 |
| 3 | 80 | 50 | 33.3 | 50 | 61 | 50 | 64 | 65 | 90 | 83 | 98 | 94.7 | 100 | 80 | 80 | 67 | 0 |
| 4 | 85 | 75 | 66.7 | 75 | 61 | 50 | 72 | 55 | 100 | 75 | 85 | 100 | 100 | 80 | 88 | 72 | 0 |
| 5 | 85 | 75 | 66.7 | 87.5 | 72 | 55 | 72 | 62 | 100 | 75 | 100 | 87.5 | 84 | 90 | 88 | 87 | 0 |
| 6 | 80 | 75 | 66.7 | 100 | 42 | 60 | 84 | 75 | 90 | 100 | 100 | 100 | 100 | 100 | 100 | 90 | 0 |
| 7 | 90 | 100 | 100 | 100 | 52 | 70 | 88 | 70 | 90 | 100 | 100 | 100 | 100 | 100 | 100 | 90 | 0 |
| 8 | 50 | 50 | 33.3 | 50 | 41 | 40 | 52 | 42 | 75 | 75 | 85 | 37.5 | 62 | 60 | 64 | 52 | 1 |
| 9 | 75 | 50 | 66.7 | 62.5 | 51 | 45 | 60 | 45 | 85 | 75 | 85 | 62.5 | 72 | 70 | 72 | 67 | 1 |
| 10 | 75 | 50 | 66.7 | 75 | 51 | 50 | 60 | 55 | 85 | 83 | 67.7 | 62.5 | 84 | 70 | 72 | 67 | 1 |
| 11 | 80 | 50 | 66.7 | 75 | 72 | 50 | 72 | 55 | 85 | 83 | 67.7 | 62.5 | 84 | 80 | 84 | 72 | 1 |
| 12 | 70 | 75 | 66.7 | 87.5 | 72 | 60 | 76 | 65 | 90 | 83 | 33.3 | 37.5 | 100 | 80 | 80 | 72 | 1 |
| 13 | 85 | 75 | 66.7 | 100 | 84 | 70 | 88 | 85 | 100 | 97 | 33.3 | 62.5 | 100 | 90 | 80 | 92 | 1 |
| 14 | 90 | 75 | 100 | 100 | 84 | 70 | 100 | 85 | 100 | 97 | 33.3 | 75 | 100 | 70 | 88 | 72 | 1 |

PF, physical functioning; RP, role physical; RE, role emotional; SF, social functioning; BP, bodily pain; VT, vitality; MH, mental health; GH, general health

^*^0=non-depressive symptoms group; 1=depressive symptoms group
